# The QseEF Two-Component System-GlmY Small RNA Regulatory Pathway Controls Swarming in Uropathogenic *Proteus mirabilis*

**DOI:** 10.3390/ijms23010487

**Published:** 2022-01-01

**Authors:** Wen-Yuan Lin, Yuan-Ju Lee, Ping-Hung Yu, Yi-Lin Tsai, Pin-Yi She, Tzung-Shian Li, Shwu-Jen Liaw

**Affiliations:** 1 Department and Graduate Institute of Clinical Laboratory Sciences and Medical Biotechnology, College of Medicine, National Taiwan University, Taipei 10048, Taiwan; f04424003@ntu.edu.tw (W.-Y.L.); joolliint@gmail.com (Y.-L.T.); R02424027@ntu.edu.tw (P.-Y.S.); qwerty7749@hotmail.com (T.-S.L.); 2 Department of Urology, National Taiwan University Hospital, Taipei 10002, Taiwan; leeyuanju@hotmail.com; 3 Department of Nursing, National Taichung University of Science and Technology, Taichung City 404348, Taiwan; oxfordocean@gmail.com; 4 Department of Laboratory Medicine, National Taiwan University Hospital, Taipei 10002, Taiwan

**Keywords:** *cheA*, *flhDC*, GlmY, *Proteus mirabilis*, QseEF, *rcsB*, swarming

## Abstract

Bacterial sensing of environmental signals through the two-component system (TCS) plays a key role in modulating virulence. In the search for the host hormone-sensing TCS, we identified a conserved *qseEGF* locus following *glmY*, a small RNA (sRNA) gene in uropathogenic *Proteus mirabilis*. Genes of *glmY-qseE-qseG-qseF* constitute an operon, and QseF binding sites were found in the *glmY* promoter region. Deletion of *glmY* or *qseF* resulted in reduced swarming motility and swarming-related phenotypes relative to the wild-type and the respective complemented strains. The *qseF* mutant had decreased *glmYqseEGF* promoter activity. Both *glmY* and *qseF* mutants exhibited decreased *flhDC* promoter activity and mRNA level, while increased *rcsB* mRNA level was observed in both mutants. Prediction by TargetRNA2 revealed *cheA* as the target of GlmY. Then, construction of the translational fusions containing various lengths of *cheA* 5′UTR for reporter assay and site-directed mutagenesis were performed to investigate the *cheA*-GlmY interaction in *cheA* activation. Notably, loss of *glmY* reduced the *cheA* mRNA level, and urea could inhibit swarming in a QseF-dependent manner. Altogether, this is the first report elucidating the underlying mechanisms for modulation of swarming motility by a QseEF-regulated sRNA GlmY, involving expression of *cheA*, *rcsB* and *flhDC* in uropathogenic *P. mirabilis*.

## 1. Introduction

*Proteus mirabilis* is an important pathogen of the urinary tract, especially in patients with indwelling urinary catheters [1]. Common strategies of pathogenesis employed by *P. mirabilis* include adherence via fimbriae [2,3], biofilm formation, flagella-mediated motility, immune modulation and urease production [4]. *P. mirabilis* exhibits a form of multicellular behavior termed swarming [5]. Swarming motility are the results of complex signal transduction and gene regulation [5]. It is generally believed that signals could be sensed and transmitted by two-component systems (TCSs). It is worth noting that the ability of *P. mirabilis* to express virulence factors is coupled to swarming differentiation [5]. In general, flagella are thought to assist in colonization and dissemination during *P. mirabilis* catheter-associated UTIs, and FlhDC is a master regulator that controls the expression of flagellum-related genes [6,7]. During swarmer cell differentiation, the expression level of *flhDC* rises dramatically [8].

Among multiple strategies for many bacteria to respond rapidly to changing environments is the regulation by the very versatile and adaptable regulatory small non-coding RNAs (sRNAs) [9,10,11]. Numerous cellular processes, such as motility [12], various stress responses and virulence factor expression are subject to the post-transcriptional control of sRNAs [9,10,11,13]. In many cases, the trans-acting sRNA mediated-regulation requires the chaperone protein Hfq to facilitate sRNA-mRNA interaction [14,15]. Two well-known ways for an sRNA to regulate translation are sequestering of ribosome binding site (rbs) and melting a secondary structure in the 5′ UTR to expose rbs, resulting in repression and activation of translation, respectively [16]. TCS and alternative σ factors have been shown to control sRNA expression [17,18]. In this regard, a PhoPQ TCS-regulated sRNA MgrR modulates expression of *eptB*, an LPS modification gene regulated by σ^E^, to affect the sensitivity to antimicrobial peptides in response to low Mg^2+^ or the presence of antimicrobial peptides [19]. In addition, a σ^E^-dependent sRNA, MicA, directly inhibited PhoPQ synthesis and consequently downregulated the PhoPQ regulons involved in pathogenicity, cell envelope composition and stress resistance [20].

Bacterial sensing of environmental signals plays a key role in regulating virulence and mediating bacterium host interactions. TCS is a common strategy used by bacteria to regulate gene expression in response to environmental cues. Enterohemorrhagic *E. coli* (EHEC) senses host hormones (epinephrine and norepinephrine) via the QseEF two-component system to activate actin polymerization and initiate formation of attaching and effacing (AE) lesions [21,22]. Deletion mutants of the *qseEF* are attenuated in virulence, as demonstrated for *E. coli*, *Citrobacter rodentium*, *Salmonella* and *Yersinia pseudotuberculosis* [23,24,25]. In addition, epinephrine can induce QseEF expression [21,24]. QseE is a sensor histidine kinase; QseF is a response regulator protein. The *qseE* and *qseF* genes are co-transcribed with *qseG*, a small outer membrane lipoprotein-encoded gene located between *qseE* and *qseF* [26]. The gene cluster *glmY-qseE-qseG-qseF* is conserved in *Enterobacteriaceae* [27]; however, previous work investigating QseEF or GlmY has been carried out mainly in EHEC and *Salmonella*. Although conserved across *E. coli* and *Salmonella*, the QseEF system has undergone specialization to regulate gene expression unique to each species.

Knowing the sensing of the stress hormones epinephrine (adrenaline) and norepinephrine (noradrenaline) through QseBC and QseEF TCSs plays an important role in modulating bacterial stress responses and virulence [24,28], we searched for the counterparts in uropathogenic *P. mirabilis* and identified QseEF homologue with high sequence identity but not QseBC. We then undertook an investigation to disclose the role of QseEF in *P. mirabilis*. Among the phenotypes screened, we found that *P. mirabilis qseF* mutant exhibited significantly reduced swarming ability and swarming-related phenotypic traits. Subsequently, the underlying mechanism of QseF-regulated swarming phenomenon was revealed to involve GlmY sRNA. For the first time, a pathway mediated by a two-component system through an sRNA was disclosed to be involved in swarming migration of uropathogenic *P. mirabilis*. The study provides a new insight into the underlying mechanisms of swarming motility in *P. mirabilis*.

## 2. Results

### 2.1. Identification of P. mirabilis qseEGF Gene Locus

**Figure 1:**
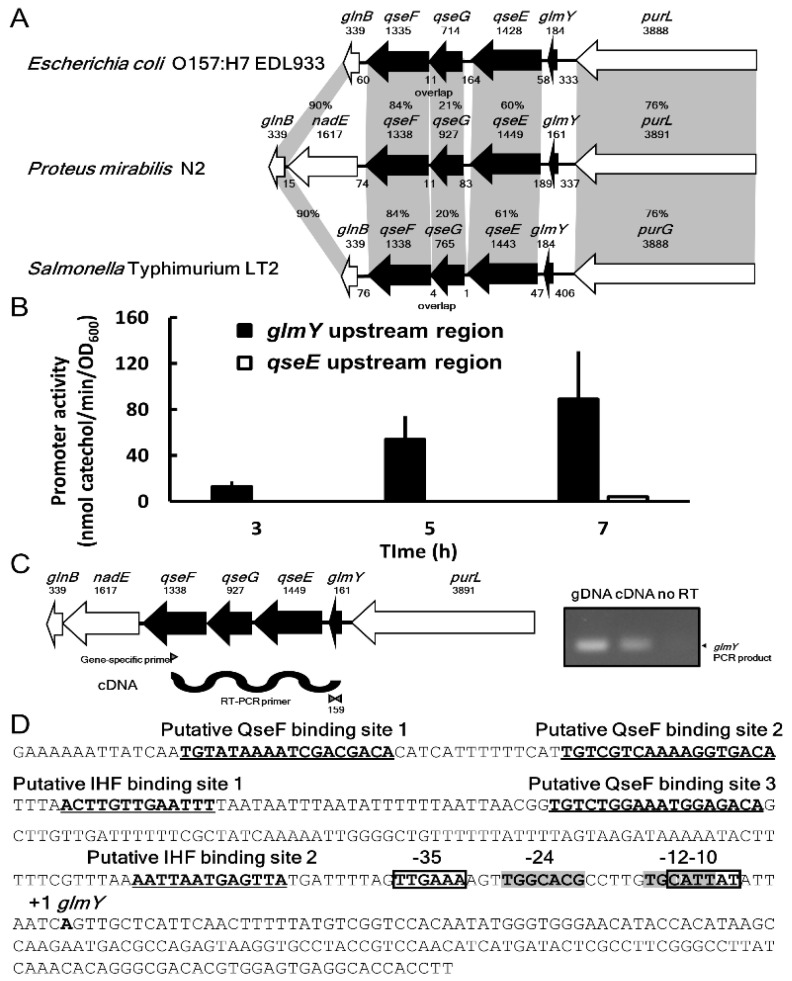
The *glmYqseEGF* gene locus in *P. mirabilis* N2. (**A**) The *P. mirabilis glmYqseEGF* gene locus corresponds to the similar locus in *E. coli* O157:H7 EDL933 and *Salmonella* Typhimurium LT2 with corresponding genes in shadows. An amino acid sequence analysis of the locus in these strains was performed using position-specific iterative BLAST. The percent amino acid identities of PurL, QseE, QseG, QseF and GlnB between *P*. *mirabilis* N2 and *E. coli* O157:H7 EDL933 or *Salmonella* Typhimurium LT2 are shown. The number above each arrow represents the gene length (bp). The intergenic space or overlap in terms of base pairs is also shown. (**B**) The promoter activities of *glmY* (−381 to −3 from putative *glmY* transcriptional start site) and *qseE* (−553 to −6 from *qseE* start codon) upstream region. (**C**) The *glmY*-*qseE-qseG-qseF* constitute an operon by gene-specific reverse transcription PCR. cDNA was synthesized by the *qseF*-specific primer and then the *glmY* DNA fragment was amplified by PCR. no RT, negative control; gDNA (genomic DNA), positive control. (**D**) The sequence of *glmYqseEGF* promoter region in *P. mirabilis* N2. The putative QseF and IHF binding sites are bold and underlined. The conserved σ^70^ and σ^54^ binding sites are indicated in boxes and shadows, respectively.

Seeing that epinephrine and norepinephrine exist in urine, we sought QseBC and QseEF homologues in uropathogenic *P. mirabilis* N2. We identified a gene locus, *purL-qseE-qseG-qseF-nadE-glnB*, whose gene product with amino acid sequence identity of around 76, 60, 20, 84 and 90% to PurL, QseE, QseG, QseF and GlnB, respectively, with *E. coli* and *Salmonella* (both lacking *nadE*) (Figure 1A). We then tested whether epinephrine or norepinephrine has any effect on phenotypic traits of *P. mirabilis*. Neither hormone in the range 0.1–400 µM altered the phenotypes assayed (motility, biofilm formation, etc.) or showed any effect on expression of *qseEF* by real time RT-PCR.

### 2.2. Identification of P. mirabilis glmY and Co-Transcription of glmY, qseE, qseG and qseF

In view of conservation of the gene cluster *glmY-glrK* (*qseE*)*-yfhG* (*qseG*)*-glrR* (*qseF*)*-glnB* in *Enterobacteriaceae* [27] and modulation of motility [12] and pathogenesis [29,30] by GlmY, we searched the bacterial small RNA database (BSRD) for *P. mirabilis* GlmY counterpart and found it located upstream of *qseE* and downstream of *purL* (Figure 1A). We first examined the promoter activity in the upstream 548-bp region of *qseE* (nucleotide −553 to −6 of QseE start codon) by the *xylE* reporter assay. No promoter activity was found (Figure 1B). Then, we tested the *glmY* promoter activity using the upstream 379-bp DNA fragment (nucleotide −381 to −3 from the putative transcription start site) of *glmY* and found the promoter activity at 3, 5 and 7 h after incubation (Figure 1B). Furthermore, we demonstrated that *glmY*, *qseE*, *qseG* and *qseF* belong to a transcript by RT (reverse transcription)-PCR using *qseF* specific primer to obtain the cDNA for amplifying the *glmY* fragment (Figure 1C). The *glmY* PCR product of 159 bp was observed, but no product was produced in the no RT control (Figure 1C). These data indicate that *glmY*, *qseE*, *qseG* and *qseF* can share the same promoter upstream of *glmY*. The lack of promoter activity in the 548-bp DNA fragment upstream *qseE* results from the fragment containing only partial *glmY* promoter DNA sequences (201/379 bp). In summary, *glmY*, *qseE*, *qseG* and *qseF* are co-transcribed from the *glmY* promoter in *P. mirabilis.* In addition, we identified one overlapped σ^70^/σ^54^, two IHF and three QseF putative binding sites in the upstream promoter region of *glmY* (Figure 1D).

### 2.3. Phenotypic Traits of glmY and qse Mutants

**Figure 2:**
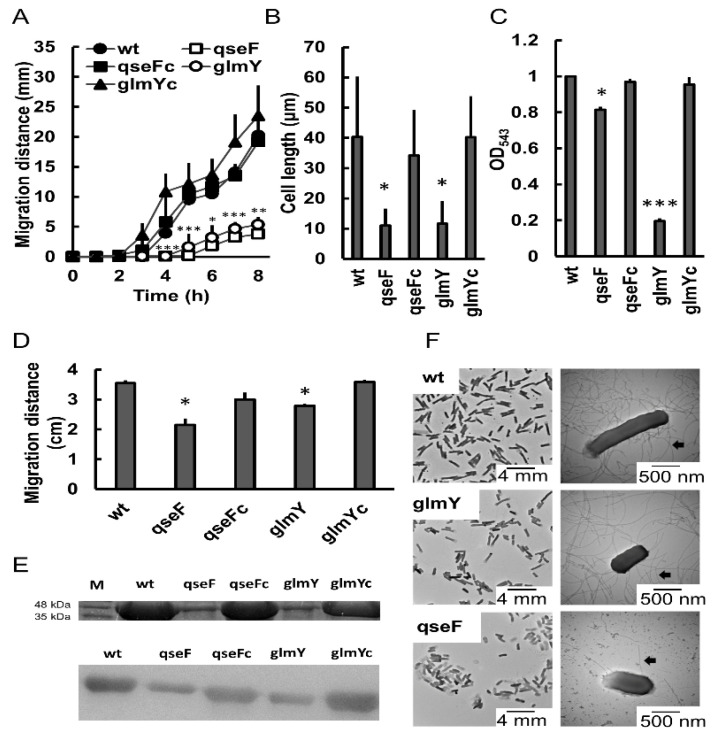
GlmY and QseF regulated swarming and related phenotypes. Swarming motility (**A**), cell length (**B**), hemolysin activity (**C**) and swimming motility (**D**) were determined in the wild-type, mutants of *qseF* and *glmY* and complemented strains. Swarming migration was monitored at 1-h intervals, and swimming motility was determined after incubation for 16 h. Cell length and hemolysin activity were determined at 5 h after incubation on the LB agar plate. The data are the averages and standard deviations of three independent experiments. Significant difference of *qseF* or *glmY* mutant from the wild-type at 4, 5, 6, 7 and 8 h is indicated (* *p* < 0.05; ** *p* < 0.01; *** *p* < 0.001 by the Student’s *t* test) in (**A**). Significant difference from the wild-type is indicated (* *p* < 0.05; *** *p* < 0.001 by the Student’s *t* test) in (**B**–**D**). (**E**) Analysis of flagellin expression by SDS-PAGE (upper panel) and Western blotting (lower panel). The flagellin level of the wild-type, mutants of *qseF* and *glmY* and complemented strains were examined at 5 h after seeding on the swarming plates by the SDS-PAGE and Western blotting as described in Materials and Methods. The representative picture of three independent experiments is shown. M, molecular weight marker. (**F**) TEM pictures of wild-type and mutants of *qseF* and *glmY*. Bacterial cultures were applied onto a carbon-coated grid, cells were stained with 1% PTA and TEM pictures were taken. Flagella are indicated by arrows. wt, wild-type; qseF, *qseF* mutant; glmY, *glmY* mutant; qseFc, *qseF*-complemented strain; glmYc, *glmY*-complemented strain.

To investigate the roles of GlmY and QseEF TCS in *P. mirabilis*, we first generated isogenic mutants of *glmY* and *qseF*. Among the phenotypes assayed, both *glmY* and *qseF* mutant strains exhibited reduced swarming and swimming abilities relative to the wild-type and respective complemented strains (Figure 2A,D). Comparable growth of the wild-type and respective complemented strains was observed, and no growth defect was found in the mutant strains compared to the wild-type (data not shown). *glmY* and *qseF* mutants migrated much slower than the wild-type and respective complemented strains during the 8-h period after inoculation on the swarming plate. Mutants lacking *qseE*, *qseG* or *qseEGF* were also constructed with no growth defect compared to the wild-type bacteria. We found similar swarming and swimming patterns among *qseF*, *qseE*, *qseG* and *qseEGF* mutants (data not shown). Therefore, we investigated the QseEGF-mediated motility using *qseF* mutant to represent QseEF malfunction in the following experiments. Significantly reduced swarming-related phenotypes, including cell differentiation, hemolysin activity and flagellin level, were observed in the *glmY* and *qseF* mutants compared to the wild-type and respective complemented strains (Figure 2B,C,E). Transmission electron microscopy (TEM) also showed *glmY* and *qseF* mutant cells were shorter and had fewer flagella than the wild-type strain (Figure 2F).

### 2.4. Downregulation of glmYqseEGF Operon by qseF Deletion

**Figure 3:**
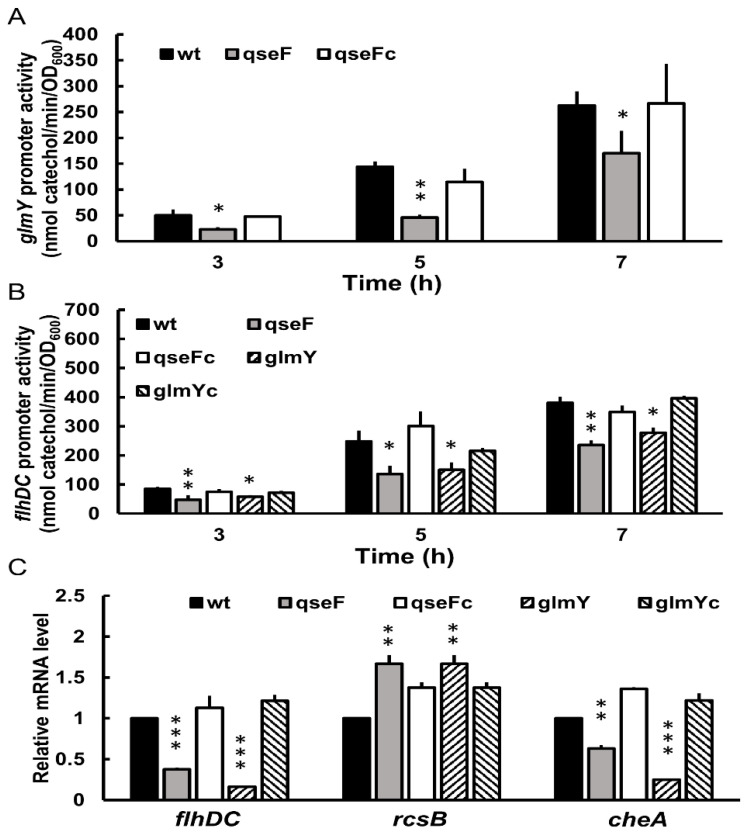
QseF-controlled GlmY regulated expression of *flhDC*, *rcsB* and *cheA*. (**A**) The *glmY* promoter activity in the wild-type, *qseF* mutant and *qseF*-complemented strain. (**B**) The *flhDC* promoter activity in the wild-type, mutants of *qseF* and *glmY* and the complemented strains. (**C**) The mRNA level of *flhDC*, *rcsB* and *cheA* in the wild-type, mutants of *qseF* and *glmY* and the complemented strains. In (**A**,**B**), bacteria cultures were spread on LB agar plate and XylE promoter activities were determined as described in Materials and Methods after incubation for 3, 5 and 7 h at 37 °C. In (**C**), the mRNA amount was measured at 5 h after inoculation on the LB agar plate. The value for the wild-type was set at 1, and other data are presented relative to this value. The data are the averages and standard deviations of three independent experiments. Significant difference from the wild-type is indicated (* *p* < 0.05; ** *p* < 0.01; *** *p* < 0.001 by the Student’s *t* test). wt, wild-type; qseF, *qseF* mutant; glmY, *glmY* mutant; qseFc, *qseF-* complemented strain; glmYc, *glmY*-complemented strain.

Based on the presence of the putative QseF binding site in the *glmYqseEGF* promoter region (Figure 1D), we examined whether *glmY* expression is under the control of QseF by the reporter assay. Just as expected, *qseF* mutant had significantly lower *glmY* promoter activity compared to the wild-type and the *qseF*-complemented strain at 3, 5, 7 h after inoculation and incubation (Figure 3A).

### 2.5. Altered Expression of flhDC, rcsB and cheA in qseF and glmY Mutants

With the notion that disruption of *qseF*, encoding a TCS transcriptional regulator QseF, impaired the swarming motility and related phenotypes in *P. mirabilis*, we thus examined the effect of *qseF* loss on *flhDC* expression by the reporter assay. The loss of *qseF* incurred significant reduction in *flhDC* promoter activity compared to the wild-type and the complemented strains at 3, 5, 7 h after inoculation (Figure 3B). Since QseF is a transcriptional regulator, we then tried to find the consensus QseF binding site (TGTCN_10_GACA) [31] in the promoter region of *flhDC* but failed. Since QseF could regulate *glmY* expression (Figure 3A) and both *qseF* and *glmY* mutants displayed decreased swarming and swimming motility (Figure 2A,D), we examined the promoter activity of *flhDC* in the *glmY* mutant. As is the case for *qseF* mutant, *glmY* mutant had a lower *flhDC* promoter activity than the wild-type and the complemented strains (Figure 3B). The mRNA level of *flhDC* in *qseF* and *glmY* mutants was consistent with the results of the reporter assay (Figure 3C).

Given the positive regulation of swarming and *flhDC* expression by both QseF and GlmY, the regulatory hierarchy from QseF to GlmY and the absence of QseF binding site in *flhDC* promoter region, we surmised that QseF may modulate swarming through GlmY to affect *flhDC* expression. How could GlmY, an sRNA, regulate both promoter activity and mRNA level of *flhDC* (Figure 3B,C)? In this respect, GlmY may modulate expression of a regulator which affects *flhDC* transcription directly and thus *flhDC* mRNA level. The direct regulation of *flhDC* mRNA level by GlmY may also contribute to the altered mRNA amount. As for *flhDC* regulator, *P. mirabilis* RcsB could modulate swarming by direct regulation of *flhDC* expression in a negative way [8,32] and *rcsB* overexpression led to a reduction of *flhDC* expression [33]. Therefore, we tested the effect of *glmY* mutation on *rcsB* mRNA level and found *glmY* mutant had increased *rcsB* mRNA level compared to the wild-type and *glmY*-complemented strain (Figure 3C). In view of regulation of *glmY* by QseF, *qseF* mutant exhibited a similar increase in *rcsB* mRNA level as the *glmY* mutant relative to the wild-type and the complemented strain (Figure 3C). On the other hand, we tried to identify whether GlmY could target *flhDC* or other mRNAs to modulate motility of *P. mirabilis*. We used the full sequence of GlmY as input to search for GlmY targets on the TargetRNA2 website. The tool did report a region in the 5′UTR of the *cheA* (but not *flhDC* or *rcsB*) as a candidate target with lower energy score and the *p*-value less than 0.05. The prediction shows interaction of GlmY (positions 17 to 28) with a 5′UTR region of *cheA* mRNA spanning −61 to −50 positions from the translation start site of *cheA* mRNA. CheA kinase, encoded by *cheA*, belongs to a family of two-component sensors responsible for bacterial chemotaxis [34]. As *P. mirabilis* GlmY positively regulated swarming (Figure 2A) and small RNAs exert positive regulation of virulence primarily at the level of mRNA stabilization [35], we assessed whether GlmY affects *cheA* mRNA level. As expected, the amount of *cheA* mRNA was reduced significantly in *glmY* and *qseF* mutants compared to the wild-type and respective complemented strains (Figure 3C).

### 2.6. GlmY Activates cheA Expression at the Post-Transcriptional Level

**Figure 4:**
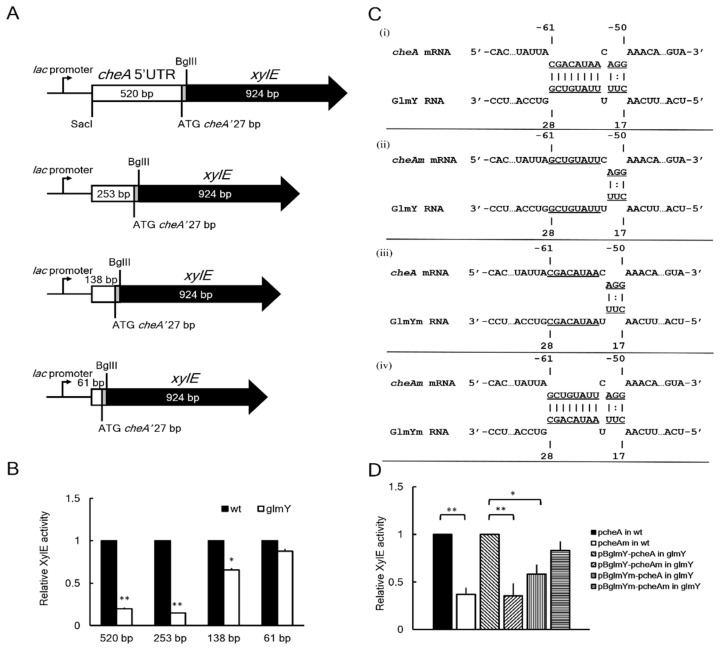
GlmY activated *cheA* expression at the post-transcriptional level. (**A**) Schematic representation of the different constructs used in the *cheA-xylE* translational reporter assay. The various lengths of *cheA* 5′UTR (520, 253, 138 and 61 bp before AUG plus 27 bp after AUG were in frame ligated to *xylE* gene to obtain the translational fusion under the control of *lac* promoter in the pGEM-T easy vector. (**B**) The effect of various lengths of *cheA* 5′UTR (as shown in (**A**)) on XylE activity of the *cheA* 5′UTR-*xylE* translational fusion in the wild-type and *glmY* mutant. The activity of XylE in the translational *cheA* 5′UTR-*xylE* reporter plasmid-transformed *P. mirabilis* strains was determined using the reporter assay at 5 h after incubation on an LB agar plate. The value obtained for the wild-type strain at a length of 5′UTR was set at 1. The data are the averages and standard deviations of three independent experiments. Significant difference from the wild-type is indicated (* *p*  < 0.05; ** *p* < 0.01 by the Student’s *t* test). (**C**) The predicted region of base-pairing between GlmY and the 5′ UTR of the *cheA* mRNA. The interaction site of *cheA* and GlmY are underlined and the substitutions present in GlmY (GlmYm) and *cheA* 5′ UTR (*cheA*m) are shown. (**i**). Wild-type GlmY and *cheA* 5′ UTR; (**ii**) wild-type GlmY and mutated *cheA* 5′ UTR; (**iii**) wild-type *cheA* 5′ UTR and mutated GlmY; (**iv**) mutated GlmY and the compensatory mutations in *cheA* 5′ UTR. (**D**) Analysis of GlmY interaction with *cheA* 5′ UTR for *cheA* expression. The *cheA* 5′UTR (253 bp)-*xylE* translational fusion plasmid (pcheA) and *glmY*-containing pBAD33 (pBglmY) were used as templates for introducing an 8-bp substitution (shown in (**C**)) into GlmY and *cheA* 5′ UTR by site-directed mutagenesis to produce pcheAm and pBglmYm, respectively. The pcheA and pcheAm were introduced into wild-type *P. mirabilis* separately, while *glmY* mutant was transformed with combinations of pBglmY-pcheA, pBglmY-pcheAm, pBglmYm-pcheA or pBglmYm-pcheAm. Then, the activities of XylE in the various *glmY* mutants (in the presence of 0.2% arabinose) and wild-types were determined at 5 h after incubation on an LB agar plate. The relative XylE activity was shown with the value of the pcheA-harbored wild-type or *glmY* mutant carrying pBglmY and pcheA set at 1, respectively. Significant difference from the wild-type or *glmY* mutant set at 1 is indicated (* *p* < 0.05; ** *p* < 0.01 by the Student’s *t* test). wt, wild-type; glmY, *glmY* mutant.

To investigate the role of interaction between GlmY and *cheA* 5′UTR in *cheA* expression and thus swarming, we generated plasmids carrying the individual *lac* promoter-driven *xylE* translational fusion containing different lengths (520, 253, 138 and 61 bp) of *cheA* 5′UTR and the first 27 bp of *cheA* ORF (Figure 4A). The individual fusion plasmid was transformed into the wild-type strain and *glmY* mutant to probe the essential region of *cheA* 5′UTR requiring GlmY for activating *cheA* translation by monitoring the *xylE* activity. While the *glmY* mutant carrying the plasmid of 520, 253 or 138 bp-5′UTR translational fusion exhibited significantly lower *xylE* activity than the wild-type strain carrying the respective fusion plasmid, albeit to a lesser extent for the 138 bp-fusion plasmid (Figure 4B), the *xylE* activity of the wild-type strain and the *glmY* mutant harboring the 61 bp-fusion plasmid was comparable (Figure 4B). The results indicate that there is no need for GlmY to activate *cheA* translation when only the 61 bp-5′UTR of *cheA* is present. The data indicate all the 520, 253 and 138-bp fragments of *cheA* 5′UTR had constrained structures requiring GlmY for releasing to activate *cheA* translation.

To further demonstrate whether GlmY affected *cheA* expression via direct base-pairing, we performed site-directed mutagenesis to inactivate the interaction of GlmY and *cheA* 5′UTR. We introduced an 8-bp mutation into the predicted pairing regions of the *cheA* 5′UTR (cheAm) and *glmY* (GlmYm) (Figure 4C) using the *cheA* 5′UTR (253 bp)-*xylE* translational fusion plasmid (pcheA) and *glmY*-containing pBAD33 (pBglmY) as templates to generate pcheAm and pBglmYm, respectively. The wild-type *P. mirabilis* harboring the pcheAm exhibited significantly lower XylE activity compared to that harboring the wild-type *cheA* 5′UTR-*xylE* plasmid (pcheA) (Figure 4D). The *glmY* mutant carrying pBglmY and pcheAm showed significantly lower XylE activity than the mutant carrying pBglmY and pcheA (0.4 vs. 1 in Figure 4D). GlmY with the 8-bp substitution (GlmYm) could not effectively enhance the expression of *cheA*-*xylE* fusion to the extent of wild-type GlmY (0.6 vs. 1) in the *glmY* mutant (Figure 4D). In addition, introduction of compensating mutations into the *cheA* 5′UTR could restore the ability of GlmYm to activate the *cheA-xylE* fusion to 80% of the wild-type GlmY and *cheA* interaction (pBglmYm-pcheAm vs. pBglmY-pcheA in Figure 4D).

These results prove the role of *cheA* 5′ UTR in interaction with GlmY and the 8-bp direct pairing between GlmY and *cheA* 5′ UTR critical for facilitating expression of *cheA*. For the first time, this work identifies *cheA* as a novel target of GlmY for modulating swarming motility of *P. mirabilis*.

### 2.7. Urea Inhibited Promoter Activity of the glmYqseEGF Operon in P. mirabilis

**Figure 5:**
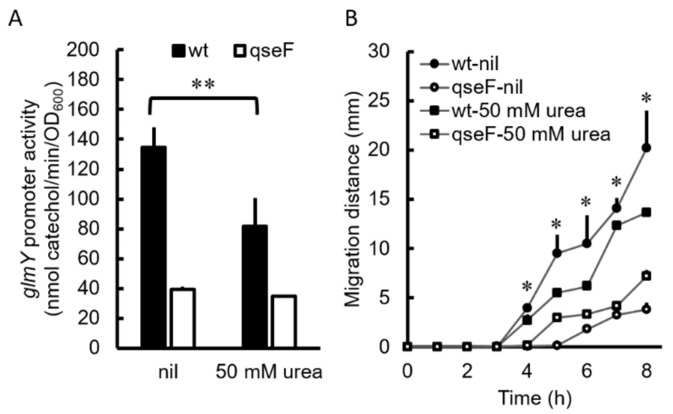
Urea inhibited *glmY* expression through QseF. (**A**) *glmY* promoter activity in the wild-type and *qseF* mutant in the presence and absence of urea. Bacterial cultures were spread on an LB agar plate with or without 50 mM of urea, and XylE promoter activities were determined at 5 h after incubation at 37 °C. (**B**) Swarming motility of the wild-type and *qseF* mutant in the presence and absence of 50 mM of urea was monitored at 1-h intervals. The data are the averages and standard deviations of three independent experiments. Significant difference between presence and absence of urea is indicated (* *p* < 0.05; ** *p* < 0.01 by the Student’s *t* test). wt, wild-type; qseF, *qseF* mutant; nil, no urea.

Based on the fact that *P. mirabilis* swarming was subject to positive regulation of the QseF-GlmY pathway, it was tempting to determine whether urea affect the expression of *glmYqseEGF* operon in the urinary tract, an environment containing a lot of urea. Therefore, the XylE activities of the *glmY-xylE* reporter plasmid-transformed wild-type and the *qseF* mutant were monitored in the presence of urea. The promoter activity of the *glmY* operon was reduced by urea at 50 mM in the wild-type but not the *qseF* mutant after incubation for 5 h on the LB agar plate (Figure 5A). We then examined the swarming motility of the wild-type and *qseF* mutant in the presence of urea or not. The swarming motility of the wild-type but not the *qseF* mutant was decreased by urea at 50 mM (Figure 5B). The results suggest that urea could be a negative signal for expression of *P. mirabilis* *glmYqseEGF* operon in the urinary tract.

## 3. Discussion

**Figure 6:**
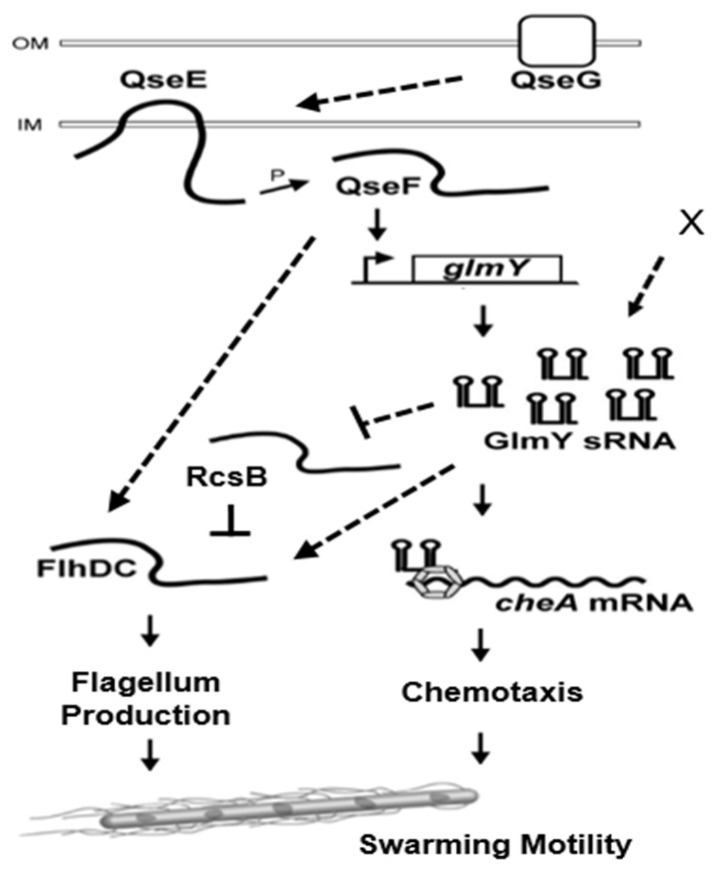
Summary of the swarming regulation by the QseEF-GlmY pathway involving expression of *rcsB*, *flhDC* and *cheA*. The sensor kinase QseE senses specific signals and transfers phosphoryl group to response regulator QseF. Then, QseF activates expression of GlmY, followed by *cheA* activation and *rcsB* inhibition. RcsB downregulation and CheA upregulation facilitate flagellum production and swarmer cell differentiation, respectively, thereby enhancing swarming motility. QseG could help activation of QseEF and unknown factor X facilitates GlmY expression. GlmY may interact with *flhDC* and modulate its expression. In addition, QseF could affect *flhDC* expression in a GlmY-independent way. Arrow, positive effect; line with a vertical bar, negative effect; dotted line, direct effect needed to be investigated.

For the first time, in this study, a TCS regulator QseF participating in modulation of swarming motility through GlmY (an sRNA) and the underlying mechanisms were revealed in uropathogenic *P. mirabilis*. GlmY regulated swarming through direct GlmY-*cheA* interaction together with GlmY-*rcsB-* and/or GlmY-mediated *flhDC* expression (Figure 6), whereby affecting swarming-related chemotaxis system and flagellum production (Figure 6). The chemotaxis system plays an essential role in flagellar function and swarm cell differentiation, thus important for *P. mirabilis* to display a vigorous swarming pattern [34,36]. Given decreased not abolished GlmY expression upon loss of *qseF* (Figure 3A), this indicates GlmY is subject to QseF-independent control. Moreover, the finding that loss of *glmY* or *qseF* resulting in a similar motility ability, expression of *flhDC* and *rcsB* and *flhDC*-associated phenotypes (cell length and flagellin level) (Figure 2 and Figure 3) indicates QseF could have alternative regulation of *flhDC* expression bypassing GlmY (Figure 6). In this regard, our preliminary data showed introduction of GlmY-expressing plasmid into *qseF* mutant could not restore *flhDC* mRNA to the wild-type level.

An ever-increasing number and variety of sRNAs are being identified to serve regulatory functions for bacteria to respond to environmental cues and thrive in diverse habitats [9,10,11,37]. *E. coli* GlmY has been coopted to modulate the expression of virulence and be involved in cellular metabolism and architecture, including for biosynthesis of LPS [38,39,40], a permeability barrier and a major virulence determinant in pathogenic bacteria [41]. GlmY fine-tunes expression of type III secretion system and its effectors to promote bacterial attachment and subsequent actin rearrangement on host cells through post-transcriptional control of EspFu and the locus-of-enterocyte-effacement (LEE) [29]. In addition, GlmY and GlmZ participate in gene expression of curli adhesion, acid resistance and also tryptophan metabolism [42]. Despite similar genomic gene arrangement of *glmYqseEGF*, there are discrepancies of the QseF and GlmY-related regulation between *P. mirabilis* and *E. coli*. First, a Rho-independent terminator exists in the end of *E. coli* GlmY [43] but not in that of *P. mirabilis*. Second, neither promoter prediction nor reporter assay showed a promoter present in the intergenic region between *glmY* and *qseE* of *P. mirabilis*, which is not the case for *E. coli*, initiating *qseE* transcription in the *glmY-qseE* intergenic region [31]. Third, *E. coli* QseEF is involved in regulating genes required for pedestal formation but not motility [21]. There was no difference in motility and flagellar expression as seen by Western blotting between the wild-type *E. coli* strain and the *qseE* mutant [21], contrary to the similar defect of both *P. mirabilis qseF* and *qseE* mutants in swarming and swimming abilities. *E. coli* *glmY* and *qseE* are independently transcribed from different promoters [31], while we demonstrated that *glmY* and *qseEGF* constitute an operon by RT-PCR assay (Figure 1C). In this regard, the presence of the conserved RNase E-cleavage motif GCCUUAU in GlmY of *P. mirabilis* [38] indicates the *glmYqseEGF* transcript could be processed to produce effective GlmY for its function.

We found swarming, swimming and swarming-related phenotypes (cell length, haemolysin activity and flagellin level) were all reduced in both *qseF* and *glmY* mutants (Figure 2). Both *flhDC* promoter activity and mRNA level were downregulated in *qseF* and *glmY* mutants (Figure 3). Because no QseF binding site in the promoter region of *flhDC*, we speculated QseF may exert its effect on *flhDC* expression through GlmY. TargetRNA2 tool, focusing its search for an sRNA-mRNA interaction in a neighborhood around the rbs of the mRNA, revealed no GlmY binding site in *flhDC* and *rcsB* mRNAs. We then extended mRNA searching by IntaRNA for GlmY-interacting sites and binding sites of −101 to −92 and −284 to −271 from AUG (both outside rbs) for *flhDC* and *rcsB*, respectively, were identified. Based on the GlmY target prediction, there are two possibilities for upregulation of *flhDC* mRNA level by GlmY. One is by direct interaction with *flhDC* mRNA; the other is indirectly through *rcsB*. It also can not be ruled out that both modes of action coexist. Further studies are needed to investigate whether GlmY directly interacts with mRNAs of *flhDC* and *rcsB*. For example, the *flhDC* (or *rcsB*) mRNA amount of the *glmY* mutant harboring wild-type or interacting site-mutated *glmY* on a plasmid will be determined to assess the GlmY-*flhDC* (or *rcsB*) mRNA interaction. It is noteworthy that overexpression of *glmY* in the same *E. coli* strain either had no effect or resulted in motility repression [12,44]. It could be due to different plasmid vectors and assay conditions used.

**Figure 7:**
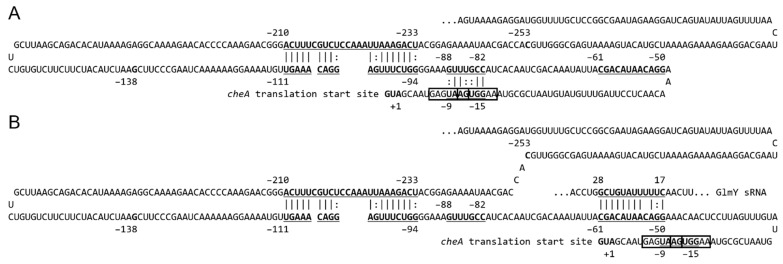
A model for regulation of *cheA* by GlmY sRNA. (**A**) The model structure for the 5′ UTR of *cheA* mRNA in the absence of GlmY. The expression of *cheA* is silenced at the post-transcriptional level by pairing −111 to −94 with −233 to −210 from AUG of *cheA* 5′ UTR and −88 to −82 from AUG with the putative ribosome binding site of *cheA*. (**B**) The putative structure for the 5′ UTR of *cheA* mRNA in the presence of GlmY. GlmY pairs with −61 to −50 from AUG of *cheA* 5′ UTR freeing the ribosome binding site and causing the *cheA* mRNA to be translated. Two overlapped putative ribosome binding sequences (AAGGUGA and GAAUGAG) are indicated in boxes, and the RNA-RNA base-pairing sequences predicted by intaRNA are underlined.

Translational fusion assay indicated that regions of −520, −253 and −138 to +27 from AUG of *cheA* mRNA contain constrained secondary structures needed to be resolved by GlmY for *cheA* translation, albeit to a lesser extent for the region of −138 to +27 (Figure 4B). To elucidate how *cheA* 5′UTR interacts with GlmY, we uploaded sequences from −253 to −138 and −138 to +27 of *cheA* mRNA to the IntaRNA webserver and interaction between regions of −233 to −211 and −111 to −94 from AUG was revealed (Figure 7). Likewise, using sequences from −138 to −61 and −61 to +27 of *cheA* mRNA as input fragments showed interaction between regions of −88 to −82 and −15 to −9 by IntaRNA (Figure 7). Inspection of the sequence of −15 to −9 disclosed the existence of two overlapped putative ribosome binding sites (rbs), aagguga (gagguga in *E. coli*) and gaaugag (gaagga in *E. coli*) for translation of long *cheA* and short *cheA* of *E. coli* [45], respectively (Figure 7). Furthermore, IntaRNA revealed interaction of *cheA* −61 to −50 from AUG with GlmY +17 to +28 (Figure 7) by using full-length GlmY and −253 to +27 of *cheA* as the input fragments. These data suggest interaction between regions of −88 to −82 and −15 to −9 from AUG could inhibit *cheA* translation by hiding rbs. Hence GlmY is required for releasing the secondary structure to assist *cheA* translation. The reason for the less extent of translation affected in the absence of *glmY* using the translational fusion comprising −138 to +27 of *cheA* compared to the −253 to +27 of *cheA* fusion could be ascribed to the interaction between regions of −233 to −211 and −111 to −94 from AUG (Figure 7), thereby affecting the ease for GlmY to uncover the rbs for translation. It is interesting to know that GlmY not only activated *cheA* translation but also maintained *cheA* mRNA level. This is in line with that sRNA-mediated stability control is the crucial element of activation of *trans*-encoded mRNAs [35].

EHEC regulated pathogenesis and motility by sensing epinephrine or norepinephrine through QseBC and QseEF two-component signaling systems [21,22]. The membrane kinase QseC autophosphorylates and phosphorylates the QseB response regulator initiating a signaling cascade that activates QseEF to trigger expression of LEE genes, leading to AE lesions on intestinal epithelial cells. In *P. mirabilis* genome, only QseEF homologue of high similarity was found, with no homologue of QseBC existing. We found QseF participated in swarming regulation and deletion of *qseE* or *qseG* also had decreased swarming motility. Previous study has revealed the phosphorylation state of QseE and QseF is governed by interaction with QseG in response to epinephrine for post-transcriptional regulation of virulence genes through GlmY [46]. In addition, QseEGF has been shown to modulate transcription of *phoPQ*, linking to the virulence regulation [47]. The similarity of the transcriptome profiles of *qseE*, *qseF* and *qseG* mutants also indicates that these proteins work together [47]. Therefore, *P. mirabilis* QseEF likely should exert functions other than swarming to affect virulence. Accordingly, our preliminary data showing *qseF* mutant had a significantly impaired ability to colonize mouse bladders and kidneys compared to the wild-type. The investigation of virulence traits such as cytotoxicity, urothelial cell invasion and survival in macrophages is underway.

We found that urea could serve as a negative signal of QseEF for swarming (Figure 5). In this way, the expression of QseF will be inhibited in the urine (rich in urea) and the role of QseF in facilitating swarming will be neglected. In view of our preliminary data showing a significant difference between the wild-type and *qseF* mutant in colonization of mouse bladders and kidneys, it is tempting to surmise there is other cues in the urine to increase expression of *qseF*. In this aspect, our unpublished transcriptome data reveal mRNA levels of fatty acid synthetic genes increase but those of degradative genes decrease in the *qseF* mutant relative to the wild-type. This indicates QseF may sense fatty acid and be involved in fatty acid metabolism. Interestingly, we found oleic acid (0.01%) is a positive signal for *qseF* expression (data not shown) and swarming motility of *P. mirabilis* [48]. The oleic acid concentration used is an attainable concentration in the urine according to the Human Metabolome Database (https://hmdb.ca/metabolites?utf8=%E2%9C%93&quantified=1&urine=1&filter=true, accessed on 29 November 2021). Given QseE is also phosphatase [23], it is reasonable to infer that the phosphatase function of QseE prevents QseF activation under the presence of urea, whereas its kinase function triggers QseF regulon expression under appropriate concentrations of oleic acid. A finely tuned balance in these opposing activities should determine the regulon response of QseF as the case of the bifunctional DevS kinase responsive to environmental oxygen in *Mycobacterium tuberculosis* [49].

Since catheter-associated UTI (CAUTI) is a major health concern, research directed at understanding the pathogenesis is warranted and should lead to improved diagnosis, prevention and treatment. *P. mirabilis* is notorious for causing CAUTIs. In this work, we demonstrated a new regulatory pathway involving an sRNA, GlmY, participating in swarming motility of *P. mirabilis* during which expression of several virulence genes is increased. It is believed that *P. mirabilis* swarming up catheters is primed to infect the urinary tract [50], so elucidating the swarming mechanisms could provide new approaches in the development of intervention strategies and facilitate the discovery of novel therapeutics.

## 4. Materials and Methods

### 4.1. Bacterial Strains, Plasmids, Reagents and Growth Conditions

The bacterial strains and plasmids used in this study are listed in Table S1 in the Supplementary Materials. The bacterial strains used are a clinical isolate from a patient of UTI (the wild-type N2), its derived mutants and respective complemented strains. All chemicals were obtained from the Sigma-Aldrich unless otherwise indicated, and primer sequences are given in Table S2. Bacteria were stored at −80 °C and routinely cultured in Luria-Bertani (LB) broth at 37 °C. The LSW^-^ agar plate [15] was used to prevent the phenotypic expression of swarming motility for selecting mutant clones and colony counting.

### 4.2. Construction of P. mirabilis Mutants and Complemented Strains

Sequences flanking the *qseF* gene was amplified by PCR using the primer pairs qseF-upF/XbaI-qseF-upR and XbaI-qseF-dnF/qseF-dnR for *qseF* mutant, and cloned into pGEM-T Easy (Promega) to generate pGqseF-up and pGqseF-dn. pGqseF-up was digested with SalI/XbaI, and the *qseF* upstream sequence-containing fragment was ligated to SalI/XbaI-digested pGqseF-dn to produce the pGqseF-updn plasmid, which contains both upstream and downstream sequences of *qseF*. A Km^r^ cassette was inserted in the XbaI-digested pGqseF-updn plasmid to generate pGqseF-updn-Km. The DNA fragment containing the Km^r^ cassette-disrupted combined upstream and downstream sequences of *qseF* was cleaved by SalI/SphI from pGqseF-updn-Km, and ligated into SalI/SphI-cleaved pUT-Km1 to generate pUTqseF-Km. For *glmY* mutant, pUTglmY-Km was constructed in a similar way except using primer pairs glmY-upF/XbaI-glmY-upR and glmY-dnF/glmY-dnR. For gene inactivation by homologous recombination, pUTqseF-Km or pUTglmY-Km was transferred to wild-type *P. mirabilis* N2 by conjugation. Transconjugants were spread on LSW^-^ plates containing tetracycline (20 μg/mL) and kanamycin (100 μg/mL), and confirmation of mutants with double-crossover events by colony PCR and Southern blot hybridization were performed. For complementation of mutants, the fragments containing full-length *qseF* gene or *glmY* was amplified by PCR using primer pairs qseF-comF/qseF-comR or glmY-comF/glmY-comR, and cloned into pGEM-T easy to generate the plasmid pGEM-qseF or pGEM-glmY. *qseF* is driven by *lac* promoter. *glmY* is driven by its own promoter (382 bp), divergent from *lac* promoter, to ensure expression of the correct transcript. pGEM-qseF or pGEM-glmY was then transformed into the respective mutant to generate the complemented strain.

### 4.3. Swarming and Swimming Assays

The swarming migration assays were performed as described previously [48]. The overnight bacterial cultures (5 µL) were inoculated onto the center of LB swarming plates containing 1.5% (wt/vol) agar, which were then incubated, and the swarming migration distance was measured by monitoring the swarm fronts of the bacterial cells at 1-h intervals. For swimming assays, the overnight culture was stabbed into the center of the swimming plates containing 0.3% agar and migration distance was recorded after incubation for 16 h at 37 °C.

### 4.4. Measurement of the Haemolysin Activity and Cell Length

The overnight bacterial cultures (120 μL) were inoculated onto the surface of LB swarming plates, which were then incubated at 37 °C for 5 h. The hemolysin activity and cell length were determined as described previously [48]. Cell-associated haemolytic activity was determined by incubation of a 20-μL cell suspension (OD_600nm_ = 0.5) in a 980-μL solution of 0.85% NaCl, 20 mM CaCl_2_ and 2% washed sheep erythrocytes at 42 °C for 15 min. After centrifugation, the amount of haemoglobin released by lysis was measured by the increase of the optical density of the assay supernatant at 543 nm.

### 4.5. Measurement of the Flagellin Level

Flagellin levels were determined as described previously by SDS-PAGE and Coomassie brilliant blue staining [15]. Western blotting was performed to confirm the flagellin band. The flagellin samples on the SDS-PAGE gel were transferred to an Hybond-P membrane (GE Healthcare, Chicago, IL, USA). The blot was incubated with mouse polyclonal antiserum against FlaA, followed by sheep anti-mouse IgG conjugated with horseradish peroxidase (GE Healthcare, Chicago, IL, USA), and then developed using enhanced chemiluminescence detection reagents (PerkinElmer, Waltham, MA, USA).

### 4.6. Transmission Electron Microscopy

Transmission electron microscopy (TEM) was performed as previously described [3] by using 1% phosphotungstic acid (PTA)-stained bacteria on a carbon-coated grid and TEM pictures were obtained with a Hitachi H-7100 electron microscope (Hitachi High-Tech America, Pleasanton, CA, USA).

### 4.7. Real Time Reverse Transcription PCR (RT-PCR)

To study the effect of *qseF* or *glmY* deletion on the mRNA amount of motility-related genes, overnight LB cultures (100 μL) of the wild-type, mutants and the complemented strains were spread on the LB agar and incubated for 5 h at 37 °C. Total RNA was extracted, and real time RT-PCR was carried out as described previously [3] to measure the mRNA level using primer pairs listed in Table S2. The levels of RNAs were normalized against housekeeping gene *gyrB* mRNA.

### 4.8. Transcriptional and Translational Reporter Assays

For transcriptional reporter assay, the promoter region of the gene was amplified by SphI and PstI-included primers and cloned into pGEM-T Easy. These promoter-containing plasmids were cut by SphI and PstI, and the promoter-containing fragment was ligated, respectively to the *xylE*-containing pACYC184-xylE digested by SphI and PstI to construct the transcriptional reporter plasmid. The XylE activity of the transcriptional reporter plasmid-transformed wild-type and mutants was measured as described previously [3]. For translational reporter assay, the 5′UTR (520, 253, 138 or 61 bp) with 27 bp coding region (5′UTR-27) of *cheA* gene was amplified by SacI and BglII-included primers and cloned into pGEM-T Easy to generate the plasmid pGcheA-5′UTR. The fragment containing full-length *xylE* gene was amplified by PCR using primer pairs BglII-xylE-F and xylE-R, and cloned into pGEM-T Easy to generate the plasmid pGxylE. pGcheA-5′UTR was digested with SacI and BglII, and the *cheA* 5′UTR-27 sequence-containing fragment was ligated to SacI/BglII-digested pGxylE to produce the translational reporter plasmid pcheA of in-frame *cheA-xylE* fusion driven by *lac* promoter. The wild-type and mutants transformed with the translational reporter plasmid were grown overnight in LB broth containing ampicillin (100 μg/mL). Then, the cultures (100 μL) were spread onto the LB agar plate and incubated for 5 h at 37 °C before the XylE activity was measured.

### 4.9. Site-Directed Mutagenesis

The translational reporter plasmid pGEM-*cheA*-*xylE* (pcheA) and the *glmY*-harboring pBAD plasmid (pBglmY)) containing mutations in the putative GlmY- and *cheA* 5′UTR-interacting site, respectively, were generated (pcheAm and pBglmYm) by a KOD-Plus-mutagenesis kit (Toyobo, Osaka, Japan) using primers listed in Table S2 according to the manufacturer’s protocol. The inverse PCR products of pcheA and pBglmY were digested by DpnI to remove the template plasmid DNA, self-ligation of PCR products was performed using T4 polynucleotide kinase and ligase and then DNA sequencing was performed to confirm the DNA sequence of the mutated sites. We introduced pcheA or pcheAm into the wild-type and compared the XylE activity to confirm the essential GlmY-interacting site in *cheA* 5′UTR. Additionally, we transformed the combinations of plasmids pBglmY-pcheA, pBglmY-pcheAm, pBglmYm-pcheA or pBglmYm-pcheAm into the *glmY* mutant and determined the XylE activity to evaluate the direct interaction of *cheA* 5′UTR and GlmY.

## Data Availability

Not applicable.

## Supplementary Materials

The following supporting information can be downloaded at: https://www.mdpi.com/article/10.3390/ijms23010487/s1.
