# Supplementary material for: The QseEF Two-Component System-GlmY Small RNA Regulatory Pathway Controls Swarming in Uropathogenic Proteus mirabilis
Source: Int J Mol Sci. 2022 Jan 1;23(1):487. doi: 10.3390/ijms23010487 (PMC8745638; doi:10.3390/ijms23010487)
Supplement: Supplementary file 1 [file ijms-23-00487-s001.zip › ijms-1510229-supplementary.pdf]

Table S1. Bacterial strains and plasmids used in this study.

Strain or plasmid	Genotype or relevant phenotype	Source
P. mirabilis		
wt	Wild-type N2; Tet^r	Clinical isolate
qseE	wt derivative; *qseE*-knockout mutant; Km^r	This study
qseF	wt derivative; *qseF*-knockout mutant; Km^r	This study
qseG	wt derivative; *qseG*-knockout mutant; Km^r	This study
qseEGF	wt derivative; *qseEGF*-knockout mutant; Km^r	This study
*qseF*c	*qseF* mutant containing pGEM-*qseF*; *qseF*-complemented strain; Amp^r	This study
glmY	wt derivative; *glmY*-knockout mutant; Km^r	This study
*glmY*c	*glmY* mutant containing pGEM-*glmY*; *glmY*-complemented strain; Amp^r	This study
E. coli		
DH5α	*fhuA2 lac(del)U169 phoA glnV44 Φ80' lacZ(del)M15 gyrA96 recA1 relA1 endA1 thi-1 hsdR17*	Invitrogen
S17-1 λ *pir*	λ *pir* lysogen of S17-1 [*thi pro* $hsdR^-$ $hsdM^+$ *recA* RP4 2-Tc::Mu-Km::Tn*7* (Tp^r Sm^r)]; permissive host able to transfer suicide plasmids requiring the Pir protein by conjugation to recipient cells	Biomedical
Plasmids		
pGEM-T Easy	High-copy TA cloning vector; Amp^r	Promega
pUT-Km1	Suicide plasmid requiring the Pir protein for replication and containing a mini-Tn*5* cassette containing Km^r gene	
pACYC184	Low-copy cloning vector, P15A replicon; Cm^r Tet^r	New England Biolabs
pBAD33	Cloning expression vector, P15A replicon; Cm^r	ATCC

Tet, tetracycline; Km, kanamycin; Cm, chloramphenicol; Amp, ampicillin; Tp, trimethoprim; Sm, streptomycin.

Table S2. Primers used in this study.

Primers	Sequence (5' to 3')	Description
qseF-upF	CAGGTCACAAAAACAGGTGTC	For *qseF* knockout. Paired with "XbaI-qseF-upR".
XbaI-qseF-upR	TCTAGACAGCCATGCACATTCTCC	
XbaI-qseF-dnF	TCTAGACGTCATCGAGCAATGTGTCG	For *qseF* knockout. Paired with "qseF-dnR".
qseF-dnR	GTTCCTCGCTCATCAAAGAC	
glmY-upF	TTTACGTGATGAGTTTGCCC	For GlmY knockout. Paired with "XbaI-glmY-upR".
XbaI-glmY-upR	TCTAGACTTGGCTTATGTGGTATG	
glmY-dnF	CATTTCTAGAGCCTGTTGC	For GlmY knockout. Paired with "glmY-dnR".
glmY-dnR	TCATGGCGTTGCGATTCTA	
qseF-comF	AGCGCAACGGTTAAACCTAC	For *qseF* complementation. Paired with "qseF-comR".
qseF-comR	CAGTTTAACTGAGCAAGTGCGAG	
glmY-comF	CCTTGGATGCGCTTATTCCGT	For GlmY complementation. Paired with "glmY-comR".
glmY-comR	AAGGTGGTGCCTCACTCCA	
flhDCrt-F	CACGAGCATGGACATTAG	For *flhDC* real-time RT-PCR. Paired with "flhDCrt-R".
flhDCrt-R	GCAGGATTGGCGGAAAGTT	
glmYrt-F	GGAACATACCACATAAGCCAAG	For *glmY* real-time RT-PCR. Paired with "glmYrt-R".
glmYrt-R	CGCTCGTCAATATTCATCTCTT	
rcsBrt-F	GCAGATGCTCTTATCACC	For *rcsB* real-time RT-PCR. Paired with "rcsBrt-R".
rcsBrt-R	CAGGCGCACCTTGTTTTA	
cheArt-F	AGATGCGAATGCGTGTACCTT	For *cheA* real-time RT-PCR. Paired with "cheArt-R".
cheArt-R	AGGCGATATCATCGGGCTTA	
gyrBrt-F	GACCCGTACGCTAAACAAC	Internal control for real-time RT-PCR. Paired with "gyrB-

		rtR”.
gyrBrt-R	AGAAATAACCGCAATCAGG	
flhDC-reF	GGGTAGATTCGCTTATTAATTCTC	For *flhDC* reporter assay. Paired with “flhDC-reR”.
flhDC-reR	CTCTTTACATCCCGTCCGAT	
glmY-reF	GCATGCCTTGGATGCGCTTATTCCG	For *glmY* reporter assay. Paired with “glmY-reR”.
glmY-reR	CTGCAGTTAATATAATGCACAAGGCGT	
BglII-xylE-F	AGATCTATGAACAAAGGTGTAATGCG	For *cheA* translational reporter assay. Paired with “xylE-R”.
xylE-R	AAGTCGTACCGGACCATCAG	
SacI-cheA-5’UTR-F	GAGCTCTATCAGGTCATACCGATGAT	For *cheA* translational reporter assay. Paired with “BglII-cheA-5’UTR-R”.
BglII-cheA-5’UTR-R	AGATCTTGTTTGATAAAACTCGG	
cheA-5’UTR 253bp	CACCAGCAATAAAAGAGGCATCAG	For *cheA* translational reporter assay. Paired with “xylE-orf-R”.
cheA-5’UTR 138bp	GCTTCCCGAATCAAAAAAGGA	For *cheA* translational reporter assay. Paired with “xylE-orf-R”.
cheA-5’UTR 61bp	CGACATAACAGGAAACAACTCC	For *cheA* translational reporter assay. Paired with “xylE-orf-R”.
xylE-orf-R	AAGTCGTACCGGACCATCAG	
cheA-mut-F	CAGCTAATATTTGTCGATTGTG	For site-direct mutagenesis. Paired with “cheA-mut-R”.
cheA-mut-R	TATTCAGGAAACAACTCCTTAG	
glmY-mut-F	AATACAGCGTCCACAATATGGGTGGGAA	For site-direct mutagenesis. Paired with “glmY-mut-R”.
glmY-mut-R	AAAGTTGAATGAGCAACTGA	
qseF-SP-RT	GCTGTATTTCTGCAAATAGC	*qseF* gene-specific RT primer
PM-qse-us-F	GTTCCTCGCTCATCAAAGAC	For *qseEGF* knockout. Paired with “PM-qse-us-R”.
PM-qse-us-R	TCTAGCGTCATCGAGCAATGTGTCG	
PM-qse-ds-F	TCTAGAAAGAGAATGAAGGTGGTGCC	For *qseEGF* knockout. Paired with “PM-qse-ds-R”.
PM-qse-ds-R	TTTACGTGATGAGTTTGCCC	

PM-qseG-us-F	GCTCAACGTGGGGATACG	For *qseG* knockout. Paired with “PM-qseG-us-R”.
PM-qseG-us-R	TCTAGACAGTTAAACCTGAACCACC	
PM-qseG-ds-F	TCTAGATTGATGCAGGGG	For *qseG* knockout. Paired with “PM-qseG-ds-R”.
PM-qseG-ds-R	GCCACGTGAATTACGCTC	
PM-qseE-us-F	GACCCAGTCACGTAGCGATAGCGGAGTGTAAGATGGCCGCTCAACAATC	For *qseE* knockout. Paired with “PM-qseE-us-R”.
PM-qseE-us-R	TTGAGACACAACGTGTCGGGGAAAAATACGCCAC	
PM-qseE-ds-F	GATGAGTTTTTCTAATCGTATTGAACTACCGCTAC	For *qseE* knockout. Paired with “PM-qseE-ds-R”.
PM-qseE-ds-R	CTGCTCTGATGCCGCATAGTTAAGCCAGTAAATAGCATCCACAGAAGC	

The recognition sequences of endonucleases are underlined.
